# The effects of curcumin on the prevention of atrial and ventricular arrhythmias and heart failure in patients with unstable angina: A randomized clinical trial

**Published:** 2019

**Authors:** Mostafa Dastani, Leila Bigdelu, Mahsa Hoseinzadeh, Hamid Reza Rahimi, Asieh Karimani, Amir Hooshang Mohammadpour, Masoumeh Salari

**Affiliations:** 1 *Department of cardiology, Faculty of medicine, Mashhad University of Medical Sciences, Mashhad, Iran*; 2 *Cardiovascular Research Center, Faculty of Medicine, Mashhad University of Medical Sciences, Mashhad, Iran*; 3 *Department of Pharmacodynamics and Toxicology, School of Pharmacy, Mashhad University of Medical Sciences, Mashhad, Iran*; 4 *Neurogenic Inflammation Research Center, Mashhad University of Medical Sciences, Mashhad, Iran*; 5 *Department of Modern Sciences & Technologies, Faculty of Medicine, Mashhad University of Medical Sciences, Mashhad, Iran*; 6 *Department of Clinical Pharmacy, School of Pharmacy, Mashhad University of Medical Sciences, Mashhad, Iran*; 7 *Pharmaceutical Research Center, Institute of Pharmaceutical Technology, Mashhad University of Medical Sciences, Mashhad, Iran*; 8 *Department of Internal Medicine, Mashhad University of Medical Sciences, Mashhad, Iran*

**Keywords:** Angina, Unstable, Arrhythmias, Cardiac, Curcumin, Heart failure, Acute coronary syndrome

## Abstract

**Objective:**

Inflammation along with oxidative stress has an important role in the pathophysiology of unstable angina which leads to acute myocardial infarction, arrhythmias and eventually heart failure. Curcumin has anti-inflammatory and anti-oxidant effects and thereby, it may reduce cardiovascular complications. This randomized controlled trial aimed to investigate the effects of curcumin on the prevention of atrial and ventricular arrhythmias and heart failure in patients with unstable angina.

**Materials and Methods:**

Forty patients with unstable angina who met the trial inclusion and exclusion criteria, participated in this double-blind randomized clinical trial. The patients were randomized into two groups: curcumin (80 mg/day for 5days) and placebo (80 mg/day for 5days). Cardiac function was evaluated by two-dimensional echocardiography devices at baseline (immediately after hospitalization) and 5 days after the onset of the trial. Atrial and ventricular arrhythmias were recorded by Holter monitors in cardiology ward, Ghaem academic hospital, Mashhad, Iran. Progression to heart failure, myocardial infarction, and pulmonary and cardiopulmonary resuscitation events as well as mortality were recorded daily throughout the study.

**Results:**

There were no significant differences between the two groups in atrial and ventricular arrhythmias (p=0.2), and other echocardiographic parameters (Ejection fraction, E, A, E/A ratio, Em, and pulmonary artery pressure) at baseline and five days after the start of the trial.

**Conclusion:**

Nanocurcumin administered at the dose of 80 mg/day for five days had no effect in the incidence of cardiovascular complications in patients with unstable angina.

## Introduction

Acute coronary syndrome (ACS) refers to a condition in which myocardial blood supply is disrupted. ACS includes ST elevation myocardial infarction (STEMI: Q-wave myocardial infarction), non-ST elevation MI (non–Q-wave myocardial infarction), and unstable angina (Gupta et al., 2013 ▶; Yeghiazarians et al., 2000 ▶). Coronary artery disease is an important cause of death worldwide. According to earlier evidence, 12.8% of total deaths occur due to ACS. In the United States, patients with ACS have an average age of 68 years (Foussas, 2015 ▶).

Unstable angina pectoris (UAP) is associated with ischemic, cardiovascular and cerebrovascular diseases. 

Among hospitalized patients with ACS, almost 26% have unstable angina (Whang et al., 2010 ▶). So, UAP has been an important subject to study over the last years. According to modern medical investigations, the origin of UAP is a local coronary artery with ischemic injuries (mostly associated with vascular endothelial lesions), platelet activation barriers, inflammation responses, vasospasm, thrombosis, and other related factors. Common treatment recommended by Western medicine involves "Antiplatelet Therapy (such as Aspirin), Antithrombin Therapy (such as Warfarin), Thrombolytic Therapy, and Conventional Antianginal Therapy (such as Beta-Blockers and Nitrates)"; nevertheless, overdosing can cause side effects such as headaches, heart palpitations and other complications (Yeghiazarians et al., 2000 ▶). According to these findings, seeking for an efficient, useful, safe, and economic way of treatment is necessary.

Curcumin (diferuloylmethane), the yellow substance found in the root of Turmeric (*Curcuma longa*) (Chuengsamarn et al., 2014 ▶; Hatcher et al., 2008 ▶; Rahimi and Oskuee, 2014 ▶; Santel et al., 2008 ▶), have many therapeutic effects. In addition, curcumin safety has been indicated by different animal trials (Anand et al., 2007 ▶; Chainani-Wu, 2003 ▶; Nabavi et al., 2014 ▶; Naik et al., 2011 ▶). Although, oral administration of curcumin for 3 months (0.5-8 g/day) had no toxic effect in patients, a higher dose (12 gr/day) seemed to be toxic (Anand et al., 2007 ▶; Hatcher et al., 2008 ▶; Sahebkar et al., 2013 ▶). Numerous studies elaborated that curcumin can target a wide range of molecules in the body; in this regard, it could act as an anti-oxidant, anti-inflammatory, anti-thrombotic, anti-carcinogenic, or a cardiovascular protective agent. Anti-inflammatory role of curcumin has a great importance among its therapeutic effects. Curcumin can reduce the expression of interleukin-6 (IL-6), tumor necrosis factor- α (TNF-α), and interleukin-1 (IL-1) by suppressing nuclear factor-κB (NF-κB). Moreover, it can inhibit mitogen-activated protein kinase (MAPK) inflammatory pathway (Shishodia et al., 2007 ▶) and thus, it plays a main role in preventing cardiovascular diseases (CVDs) (Wongcharoen and Phrommintikul, 2009 ▶).

Srivastava et al (1985) ▶ were among the first researchers who assessed curcumin efficacy on CVDs (Srivastava et al., 1985 ▶). 

Two other studies were also conducted in this field. These studies reported the therapeutic effect of curcumin on cardiac hypertrophy (Mirzabeigi et al., 2015 ▶; Tsimikas and I Miller, 2011 ▶). 

Some investigators found that curcumin can reduce very low density lipoprotein (VLDL), low density lipoprotein (LDL), cholesterol and serum triglyceride (TG) in coronary artery disease (Mirzabeigi et al., 2015 ▶). A number of studies was conducted to evaluate the correlation between the risk of CVDs and inflammation (Libby, 2006 ▶;Mason and Libby, 2014 ▶; Tsimikas and I Miller, 2011 ▶). In one study, Alwiet al. (2016**)** reported decreased levels of high-sensitivity C-reactive protein (hsCPR) after seven days of using low doses of curcumin (Alwi et al., 2016 ▶). On the contrary, another study found that curcumin had no effect on this inflammatory factor (Mirzabeigi et al., 2015 ▶).

In another study, the authors showed that curcumin can prevent and treat different pro-inflammatory chronic diseases. As a result, curcumin may prevent these disorders by stopping inflammatory processes (Wongcharoen and Phrommintikul, 2009 ▶). However, there are few studies on the effects of curcumin against the inflammatory responses in cardiovascular diseases. These studies were mostly animal experiments and human *in-vitro* studies (ABE et al., 1999 ▶; Jobin et al., 1999 ▶; Kang et al., 1999a ▶; Kang et al., 1999b ▶).

Due to contradictory results about the effect of curcumin on CVDs, there is a great need for more investigations. This randomized clinical trial aimed to investigate the effects of curcumin on the prevention of atrial and ventricular arrhythmias and heart failure in patients with unstable angina.

## Materials and Methods

**Study design**

A randomized, double-blind, clinical trial was designed to evaluate the effects of curcumin on the prevention of atrial and ventricular arrhythmias and heart failure in patients with UAP. This study was conducted at the Cardiology ward, Ghaem academic hospital, Mashhad University of Medical Sciences, Mashhad, Iran. This study began in September 2014 and ended in May 2015. Forty patients who met the inclusion and exclusion criteria, were recruited and an informed consent was obtained.

The study was approved by the Institutional Ethics Committee of Mashhad University of Medical Sciences, Mashhad, Iran. This trial was registered in the Iranian Registry of Clinical Trial (IRCT2013102315122N1). Moreover, it conforms to the CONSORT guidelines. 

**Patients**

Considering that no clinical study had been carried out in this context, it was not possible to determine the sample size based on a previous report; therefore, based on inclusion and exclusion criteria, 40 patients were selected and enrolled in the study. As a pilot study, the results of this experiment could be utilized for calculating sample size in further investigations.

Patients accepted their enrollment in the study by signing an informed consent form. Patients over 20 years old, diagnosed with unstable angina (based on New York Heart Association (NYHA) 2013 guideline), were included. Patients with renal and hepatic failure, acute or chronic infections, malignancies, chronic inflammatory diseases, history of arrhythmia, heart failure and those who required PCI (percutaneous coronary intervention) emergency, or were allergic to curcumin or immunosuppressive and anti-inflammatory drugs except statins, and pregnant or lactating women were all excluded.

The diagnosis of unstable angina was made by an experienced cardiologist. At first, all the patients were examined by an internist and evaluated for inclusion and exclusion criteria. Patients diagnosed with unstable angina were visited daily by a cardiologist and arrhythmia was evaluated during the study. The cardiac arrhythmias were recorded by cardiac monitoring during their hospitalization in the CCU. On the first and fifth days, an echocardiographic evaluation was performed by a second cardiologist who was blinded to the study protocol.

**Figure 1:**
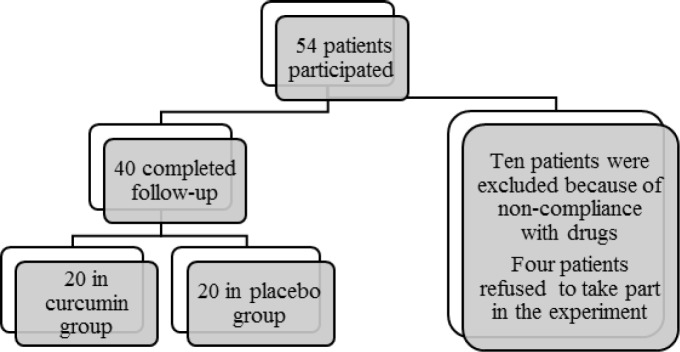
Study participation diagram

**Intervention**

Patients were randomly allocated into two groups by simple randomization based on computer-generated random numbers. Neither patients nor investigators knew that who will be allocated to which group. The assessor and the statistical analyst were also blinded to the treatment allocation. Each group consisted of 20 patients (Figure1).

Since curcumin has a lipophilic nature, its absorption is very low (Anand et al., 2007 ▶; Hani and Shivakumar, 2014 ▶; Hatcher et al., 2008 ▶). In this study, soft gelatin capsules containing nanocurcumin with the brand name of SinaCurcumin™ were prescribed. SinaCurcumin is a certified curcuminoid product in Iran (IRC: 1228225765) extracted from the dried rhizomes of *Curcuma longa* L. (turmeric) and comprises curcumin, desmethoxycurcumin, and bisdemethoxycurcumin. These components are all together known as the C3 complex. Each soft gelatin capsule of SinaCurcumin possesses 80 mg curcuminoid as nanomicelles. The encapsulation adequacy of curcuminoid in nanomicelles is almost 100%. The mean diameter of nanomicelles is around 10 nm, as measured by dynamic light scattering. The oral absorption of SinaCurcumin was at least 50 times greater than the conventional powder of curcumin, in mice (Ahmadi et al., 2018 ▶; Kakkar et al., 2011 ▶; Rahimi et al., 2016a ▶; Rahimi et al., 2016b ▶).

Based on the above-noted findings and similar investigations done before (Rahimi et al., 2016a ▶), nanocurcumin (80 mg) was used in this study.

For the second group, the placebo had a exactly similar appearance as curcumin; however, it contained lactose instead of curcumin. Patients continued to take previously prescribed drugs, including nitrates, beta blockers, angiotensin inhibitors, statin, aspirin and Plavix during the study.

The intervention group received 80 mg curcumin capsule daily for 5 days and the control group daily received a placebo capsule. Patients were advised to take the drug after the meal to avoid possible digestive complications. All patients hospitalized and monitored for five days in the CCU of Ghaem hospital. The heart function was evaluated immediately after hospitalization (day 0) and at the end of the study (day 5) by echocardiography, (SIEMENS, Acusons SC2000) and atrial and ventricular arrhythmias were recorded by Cardiofax C, Nihon Kohden. Heart failure, myocardial infarction, the rate of cardiopulmonary resuscitation, and eventually the mortality rate were all noted throughout the experiment.

**Criteria of effectiveness**

*Evaluating the incidence of arrhythmia*

Patients’ electrocardiograms were taken and the occurrence of atrial and ventricular arrhythmias was evaluated during five days (day 0-5). Arrhythmias were recorded by the nurses who were checking cardiac monitoring of patients constantly in CCU.

*Evaluating echo and factors*

Echocardiograms were obtained using SIEMENS, Acusons SC2000 with a 2.5-3.5 MHz probe by experienced echocardiologist according to last echocardiographic guideline. The sizes of the left ventricle and left atrium were measured in the parasternal view in M mode. The left ventricular ejection fraction was calculated in the apical two- and four-chamber views in two-dimensional mode using the Simpson’s rule. 

The left ventricular diastolic function was evaluated using the mitral inflow velocities (E, A) pattern, which usually can be defined as various stages of diastolic dysfunction.

Myocardial relaxation was also assessed by tissue Doppler imaging. Both of the above methods were employed to grade diastolic dysfunction. The ratio of transmitral Doppler early filling velocity to tissue Doppler early diastolic mitral annular velocity (E/Em) was utilized to estimate the filling pressure. The pulmonary capillary wedge pressure will be ≥20 mmHg if the E/Em is ≥15 and will be normal if the E/Em is <8. When the E/Em is between 8 and 15, pulmonary vein flow velocities and Valsalva maneuver were used to estimate the pulmonary capillary wedge pressure. The pulmonary arterial pressure (PAP) was measured based on echocardiographic parameters.

The primary endpoint of this study was progression towards heart failure and ST segment elevation myocardial infarction (STEMI)*.*

The secondary endpoint was the effect of curcumin on electrophysiology and mechanical function of the heart, based on Holter monitoring and echocardiography.

**Statistical analysis**

Kolmogorov-Smirnov (KS) test was used to assess normal distribution of data and Levene test (Nordstokke et al., 2011 ▶) was used to evaluate homogeneity of variance. Subsequently, to compare Confidence Intervals (CI), an independent T test for variables with normal distribution or Mann-Whitney test for variables with abnormal distribution was done. A chi-square test was also used to compare prevalence in two groups. A significance level of p<0.05 was considered in all tests. Data analysis was done by using the SPSS, version 16.

## Results

**Patient characteristics**

Demographic characteristics and cardiovascular risk factors of study population are listed in Table 2. There were no statistically significant differences in various parameters between the two groups (p>0.05).

**Table1:** Approaches used for diagnosis of different types of arrhythmia

**Types of arrhythmia**	**Diagnoses**
**Ventricular tachycardia**	P-wave may be seen, rate 100-150/min, regular rhythm abnormal contour (>0.12 Sec) Non-sustained VT: VT lasting shorter than 30 seconds. Sustained VT: VT lasting longer than 30 seconds or with hemodynamic collapse.
**Ventricular fibrillation**	P-wave: difficult to see QRS complex: rate 400-600/min, grossly irregular, baseline undulation no
**Atrial premature complex**	P-wave: P-waves different from regular P-waves and appear sooner than them Narrow QRS complex

**Table 2:** Demographic characteristics of curcumin and placebo groups

**Variables**	**Placebo** **n=20 (%)**	**Curcumin** **n=20 (%)**	**Total** **n=40 (%)**	**P-value**
**Age, year** 1	63.0±12.31	59.63±10.55	61.31±11.40	0.412
**Gender**				
**Male**	42.9	41.2	42.1	0.917
**Female **	57.1	58.8	57.9	
**UDM**	37.5	25	31.3	0.446
**Smoking** 2	25.0	31.3	28.1	>0.99
**Drug abuse** 3	12.5	0	6.3	0.484
**Hypertension**	62.5	68.8	65.6	0.710
**Familial history of CVD**	6.3	12.5	9.4	>0.99

UDM, uncontrolled diabetes mellitus

Data are expressed as mean±SD and percentage

Chi-squared test

1 T-test

2, 3 Fisher's exact test

**Comparison of the effect of curcumin on the incidence of cardiac arrhythmias in the drug and placebo groups**

Table 3 demonstrates that premature ventricular complexes, short-term ventricular tachycardia, and arrhythmia were not significantly different between placebo and curcumin groups (p>0.05).

**Table 3:** Effects of curcumin on prevalence of different arrhythmias (percentage) in placebo and curcumin groups

**Variables**	**Placebo** **n=20 (%)**	**Curcumin** **n=20 (%)**	**P-value**
**Premature ventricular complexes**	5	20	0.292
**Short-term ventricular tachycardia**	0	7.7	0.406
**Frequent atrial premature complexes**	0	7.7	0.406
**Atrial premature complex arrhythmia**	5	0	>0.99
**Arrhythmia**	9.5	29.4	0.207

**Comparison of the effect of curcumin on echocardiographic parameters in drug and placebo groups**

There were no significant differences in various echocardiographic parameters between drug and placebo groups (p>0.05).

**Table 4:** Echocardiographic changes in placebo and curcumin groups on day 0 and 5 (mean±SD)

**EF**			
	Day 0	52.94±9.36	0.885
	Day 5	52.35±9.20	0.779
	EF_5_-EF_0_	-0.58± 2.42	0.738
**E**			
	Day 0	72.18±24.41	0.459
	Day 5	76.47±27.32	0.074
	E_5_-E_0_	4.29±12.04	0.227
**A**			
	Day 0	78.82±24.35	0.497
	Day 5	78.71±25.93	0.906
	A_5_-A_0_	-0.12±7.99	0.338
**E/A**			
	Day 0	84.00±52.31	0.326
	Day 5	112.67±67.24	0.168
	E/A_5_-E/A_0_	28.67±70.82	0.493
**Em**			
	Day 0	62.00±17.55	0.399
	Day 5	61.53±14.52	0.622
	Em_5_-Em_0_	-0.47±10.32	0.726
**DT**			
	Day 0	242.53±52.79	0.636
	Day 5	240.00±63.27	0.552
	DT_5_-DT_0_	-2.53±40.76	0.767
**PAP**			
	Day 0	29.75±5.09	0.023
	Day 5	29.13±2.90	0.050
	PAP_5_-PAP_0_	-2.29±13.01	0.820

EF, ejection fraction; E, the first stage of ventricular filling in Doppler echocardiography; A, atrial contraction stage in Doppler echocardiography; E/A, left ventricular filling with blood pumping during atrial contraction; Em, first stage of ventricular filling in tissue Doppler echocardiography; DT, necessary time to reduce left ventricular rapid filling flow; PAP, pulmonary artery pressure.

It was found that pulmonary artery pressure (PAP) was substantially different in the two groups at the onset of the study (27.75±5.09 vs. 26.28±3.25, p=0. 023). After five days, the difference between the two groups was slightly significant (29.13±2.90 vs. 26.29±4.64, p=0. 050). Other factors mentioned in Table 4 did not vary between the study groups (p>0.05).

**Table:** Definition of echocardiographic factors

**E**	First stage of ventricular filling in Doppler echocardiography Indicating blood velocity through Mitral Valve
**A**	Atrial contraction stage in Doppler echocardiography
**E/A RATIO**	Indicating left ventricle filling with blood pumping during atrial contraction
**Em**	The first stage of ventricular filling in tissue Doppler echocardiography
**PAP**	Pulmonary artery pressure Indicating left ventricular systolic and diastolic function
**DT**	Necessary time to reduce left ventricular rapid filling flow
**LVEF**	Left ventricular ejection fraction that shows left ventricular systolic function

## Discussion

Contrary to our expectations, curcumin failed to reduce cardiovascular complications such as arrhythmias and heart failure in unstable angina. Echocardiographic studies showed no significant difference between placebo and curcumin groups in left ventricular ejection fraction (LVEF) and echocardiographic factors.

Several studies have found a strong relationship between the risk of cardiovascular diseases and inflammation (Libby, 2006 ▶; Mason and Libby, 2014 ▶; Tsimikas and I Miller, 2011 ▶). Patients with unstable angina pectoris have elevated amounts of highly sensitive C - reactive protein (hsCRP) that is an inflammatory factor (Haverkate et al., 1997 ▶; Liuzzo et al., 1999 ▶; Yamashita et al., 2003 ▶). 

In this regard, some researchers evaluated anti-inflammatory effects of curcumin on cardiovascular diseases (Chen et al., 2013 ▶; Duan et al., 2012 ▶; Mirzabeigi et al., 2015 ▶).

Mirzabeigi et al. (2015) ▶ conducted a randomized controlled trial (RCT) to assess the effects of curcumin on some cardiovascular risk factors in patients with coronary artery disease (CAD). The patients were divided into two groups which received either curcumin or placebo capsules (500 mg), four times a day for 8 weeks. The results demonstrated that curcumin improved several lipid profile components, but had no considerable effect on inflammatory markers (hsCRP) in these patients (Mirzabeigi et al., 2015 ▶). Also, another double-blinded randomized clinical trial conducted by Khosravi et al. (2016) ▶ in 35 chronic renal failure patients showed that curcumin (500 mg every 8 hours for 6 weeks) had no effect on improving LV function and LVEF (Khosravi et al., 2016 ▶). These results may support our findings that curcumin is rather ineffective on unstable angina.

In contrast to earlier findings, in another double-blinded clinical trial, researchers randomized 75 ACS patients into three intervention groups (15 patients in each group) taking different doses of curcumin (15, 30, and 60 mg three times a day), and the placebo group (30 patients). It was found that lower doses of curcumin could significantly decrease hsCRP level after seven days of use (Alwi et al., 2016 ▶). Sandur et al. (2007) found that curcumin may have contradictory effects at various concentrations. It has been stated that curcumin can have both antioxidant and pro-oxidant activities.

Many cardiovascular diseases such as atrial arrhythmias are mainly caused by inflammatory processes (Schoonderwoerd et al., 2008 ▶). Considering the anti-inflammatory effects of curcumin, it may be beneficial to prevent these disorders. Our study was the first to examine the effects of curcumin on atrial and ventricular arrhythmias. Contrary to our expectations, the results of our study failed to show a meaningful association between curcumin and arrhythmia. 

Inflammation increases severely in acute coronary syndrome, therefore, it might be better to give higher doses of curcumin to achieve better results. Due to the fact that curcumin efficacy remains for a short time, we should have increased the frequency of curcumin administration. Curcumin regulates kinases, many transcription factors, cytokines, and growth factors (Rahimi et al., 2016a ▶). Considering its role in gene transcription, more follow-up may be required to investigate the effects of this drug.

This information can be used to develop investigations on finding affordable medicines with low side effects such as curcumin, to treat complications of cardiovascular diseases.

In general, it seems that after the occurrence of unstable angina, curcumin capsule (80 mg/ day for 5 days) had no effect on the incidence of cardiovascular complications in patients. This randomized controlled trial has extended our knowledge about curcumin effects on ACS.
